# Family network typologies of adults with intellectual disability: Associations with psychological outcomes

**DOI:** 10.1111/jar.12786

**Published:** 2020-07-25

**Authors:** Tess Tournier, Alexander H. C. Hendriks, Andrew Jahoda, Richard P. Hastings, Sanne A. H. Giesbers, Ad A. Vermulst, Petri J. C. M. Embregts

**Affiliations:** ^1^ Tranzo Tilburg School of Social and Behavioral Sciences Tilburg University Tilburg The Netherlands; ^2^ ASVZ Sliedrecht The Netherlands; ^3^ School of Pedagogical and Educational Science Faculty of Social Sciences Radboud University Nijmegen Nijmegen The Netherlands; ^4^ Institute of Health and Wellbeing University of Glasgow Glasgow UK; ^5^ Centre for Educational Development, Appraisal and Research University of Warwick Coventry UK; ^6^ Centre for Developmental Psychiatry and Psychology Department of Psychiatry School of Clinical Sciences at Monash Health Monash University Clayton Vic. Australia; ^7^ GGZ (Mental Health Care) Oost Brabant Boekel The Netherlands

**Keywords:** family relations, latent class analysis, social capital, social networks, social support

## Abstract

**Background:**

Based on self‐reported social capital, different typologies of family networks of people with intellectual disabilities were examined. Associations between behavioural and emotional problems or well‐being and typologies were investigated.

**Method:**

137 participants with mild intellectual disability were interviewed using the Family Network Method‐Intellectual Disability to assess their emotionally supportive family relationships. Data on participants’ well‐being and behavioural and emotional problems were also gathered. Latent class analysis was used to identify family typologies based on social network measures.

**Results:**

Four distinguishable typologies were identified, two supportive and two less supportive. A small association was found with behavioural and emotional problems and one of the supportive typologies. Associations with constructs of well‐being were found for both supportive and less supportive typologies.

**Conclusions:**

A variety of family types were found, with implications for sensitive professional support.

## 1 INTRODUCTION

Supportive social relationships, including those of family, may have a positive influence on health and well‐being of people with and without intellectual disabilities (Antonucci, 2001; Scott & Havercamp, 2018). Relationships serve many functions, including providing an outlet for frustrations and fears and giving assistance and encouragement in times of difficulty (Scott & Havercamp, 2014). In the field of intellectual disability, researchers have examined the association between various outcomes and social networks, mainly analysing the effects of particular aspects of a social network such as total network size (Lippold & Burns, 2009) and the amount of perceived support (Forrester‐Jones et al., 2006). However, the structures in which these social relationships are embedded matters (Faber & Wasserman, 2002). The degree to which an individual is integrated into a broad social network is directly linked with well‐being and mental health (Cohen & Wills, 1985; Sapin, Widmer, & Iglesias, 2016). As such, social networks are a significant source of social capital (Furstenberg & Kaplan, 2004). Social capital theory describes the possession of a durable social network as a source of socially supportive relationships (Bourdieu, 1986). Based on this theory, treating network characteristics as individual dimensions (e.g. size of a network) will fail to capture the multi‐dimensional nature of networks (e.g. a dense network with many reciprocal supports; Fiori, Antonucci, & Cortina, 2006).

Most research about family networks of people with intellectual disability has only examined the person with an intellectual disability’s view of their own relationships with other family members and not the (reciprocal) relations between all network members (Lippold & Burns, 2009; Robertson et al., 2001; Van Asselt‐Goverts, Embregts, & Hendriks, 2013). There is a small qualitative literature about people with intellectual disabilities’ perspectives on mutual care, between them and their family members. In one study (Walmsley, 1996), 22 adults with an intellectual disability were interviewed about their lives, and giving and receiving care. Results suggested that the participants did not view themselves as being dependent on family care. Instead, they saw themselves as having family roles which allowed for a sense of reciprocity and mutuality. Williams and Robinson (2001) compared the perspectives of 40 parents with those of 45 (young) adults with an intellectual disability. Based on the interviews, they found that many people with intellectual disabilities and their parents did not feel that there was mutual support. Parents generally defined themselves as carers who took responsibility and exercised control. Both of these studies suggest that a more holistic approach is required, taking into account the complex web of interdependence within families; a model that recognizes mutually supportive relationships and considers the resources needed by the whole family. To capture the multi‐dimensional nature of family networks, approaches such as the Family Network Method are required (Widmer, Aeby, & Sapin, 2013). The Family Network Method is an instrument that maps who the participant considers to be their family members. In addition, it assesses how the participant perceives the relationships between these family members (Widmer, Kempf, Sapin, & Galli‐Carminati, 2013). This method has been adapted by Giesbers et al. (2019) as the Family Network Method‐Intellectual Disability (FNM‐ID) to ensure it is accessible and meaningful for use in the population of individuals who have an intellectual disability.

Initial results using the FNM‐ID showed that there is likely to be considerable variation in the perceived family networks of people with intellectual disabilities, in terms of size and (reciprocal) emotional support (Giesbers et al., 2020). Examining family typologies can have practical implications, as different types of families might require different professional support. There has been very little research about family network typologies in the field of intellectual disabilities. The few studies published have been focused on parents’ perceptions instead of the perception of the person with intellectual disabilities themselves (Mink, Meyers, & Nihira, 1984; Mink, Nihara, & Meyers, 2002). One previous study did take the perspectives of people with intellectual disability into account, to identify typologies of their family networks. Widmer, Kempf, et al. (2013) explored the different family roles instead of the significant emotional support they provide. Widmer, Kempf, et al. (2013) performed a cluster analysis and described four family configurations: professional, kinship, nuclear and friendship family configurations. However, Widmer, Kempf, et al. (2013) did not distinguish who is providing or receiving emotional support to/from the person with intellectual disability, which is the essence of social capital (Bourdieu, 1986).

Given the lack of family network typology studies, based on self‐reports of people with intellectual disabilities, the current study examined whether different typologies of perceived family networks can be distinguished in terms of emotional support. Previous research has also shown associations between dimensions of family‐based social capital and the behaviour problems (McPherson et al., 2014) and living situation (Widmer, Kempf, et al., 2013) of people with intellectual disabilities. Therefore, a secondary aim of the current study was to examine associations between types of family networks and the personal characteristics of the individuals with intellectual disabilities, their behavioural and emotional problems, and their well‐being.

## 2 METHOD

### 2.1 Participants

The 137 participants had a mean age of 28.2 years (SD = 6.16, range 18–40); 56.2% of the participants were male, 92.0% had a [COUNTRY] cultural background, and 44.9% were officially diagnosed by a certified clinician with a psychiatric or developmental disorder, with autism the most common category (24.1%). Most of the participants lived in a facility in the community (83.9%), and the others lived in a residential facility (see Table 1). The mean age of staff was 41.96 years (range 23‐63), 26 were male (19.0%), the average work experience was 18.62 years (range 3‐45), and 92.7% (*n* = 127) had received specific training in the field of social work or health care, of which 63.8% (*n* = 81) involved an intermediate vocational training and 35.4% (*n* = 45) higher professional education and training.

**TABLE 1:** Demographic information for the 137 participants with intellectual disabilities

	*N*	Per cent (%)	Mean	*SD*
Sex				
Male	77	56.2		
Female	60	43.8		
Age in years			28.20	6.16
Place of residence				
Community	115	83.9		
Residential	22	16.1		
Living condition				
Individually	47	34.3		
With others	90	65.7		
Cultural Background				
[COUNTRY]	126	92.0		
Other	11	8.0		

### 2.2 Procedure

After approval by the Ethics Committee of [NAME UNIVERSITY] (EC‐2015.46), participants were randomly selected using a stratified sampling procedure within five service providers for people with intellectual disabilities in the [NAME COUNTRY]. For each service provider, the total number of people with intellectual disabilities who met the inclusion criteria was identified. Then, a sample of 10% of the population who met the inclusion criteria of each service provider were selected for the study. Inclusion criteria to participate in the cross‐sectional study were as follows: (a) age between 18 and 40 years; (b) mild intellectual disability; and (c) support at least once a week by paid support staff for at least 6 months. Participants were approached through their key support worker. A total of 354 individuals were selected randomly and invited to participate, and 42.4% agreed to take part in the study (*N* = 150). Reasons for non‐participation included no interest (*n* = 117, 57.4%) and objections from relatives or guardians (*n* = 21, 10.3%). For some individuals, the support worker or psychologist advised against participation in the study (*n* = 66, 32.4%).

When the person with intellectual disability agreed to participate, an appointment was made. Depending on participant preference, face‐to‐face interviews were carried out at the participants’ homes or at the service providers’ offices. The researcher carefully explained the purpose of the study, the procedure and the confidentiality of the information. A standard consent procedure (Arscott, Dagnan, & Kroese, 1998) was then followed to assess the capacity of the participant to give consent to take part in the research. Participants were given a written and verbal overview of the research project, and the researcher asked them the five questions developed by Arscott et al. (1998), to determine whether they could recall information about the study. When the participant demonstrated sufficient recall, a written consent form was signed. If the participant could not answer the questions, the researcher explained the project again in more accessible terms, until the participant was able to understand the key aspects of the research project. Once consent was obtained, Wechsler Adult Intelligence Scale‐IV subtests were carried out. Demographic information was obtained from the participants, and the Personal Wellbeing Index‐Intellectual Disability was completed as a questionnaire. Then, the FNM‐ID was used to interview the participant about their family network. The administration of the FNM‐ID was audio‐recorded if the participant gave permission.

After the interview, participants were given a ten euros in recompense for their time. Eleven participants were excluded because their scores on the cognitive assessments were above or below the mild intellectual disability range. Two individuals were excluded because they were not able to answer the questions, leaving a total of 137 participants.

With the participant’s consent, their key support worker was interviewed to obtain information about the participants’ behavioural and emotional problems and to check additional information about officially diagnosed psychiatric or developmental disorders obtained from personal records.

### 2.3 Measures

#### 2.3.1 Family networks

The Family Network Method‐Intellectual Disability (FNM‐ID) was used to measure the family networks of people with intellectual disability. The FNM‐ID makes it possible to analyse emotionally supportive relationships, by asking participants to estimate relationships among all their family members. Emotional support refers to a belief that love and caring, sympathy and understanding, and/or esteem and value are available from significant others (Thoits, 1995). The instrument is composed of multiple steps; participants are first asked to provide a list of all individuals whom they consider to be a family member at the time of the interview. The term “family” is deliberately left open to allow the participants to apply their own definitions. Subsequently, participants are asked to list all family members that are significant to them from the listed family members. Finally, they are asked to describe which family members provide emotional support to the participant and to each other from the list of family members, by asking them the question: “If X is feeling ‘out of sorts’, who is there for X?” (i.e. X represents each individual included the participant’s family configuration, considered in turn). Socio‐demographic information on each listed family member is collected, as well as information on the nature of the family tie, the duration of the relationship (if not a relative) and the frequency of contact.

To characterize the family networks of individuals with intellectual disability, seven social network measures are computed from the FNM‐ID (Table 2), which are related to a social capital theoretical perspective (Sapin et al., 2016; Widmer, Kempf‐Constantin, & Carminati, 2010) and are of interest in terms of the lives of people with intellectual disability.

**TABLE 2:** Overview of the computed social network measures

Type of network	Network measures	Definition
Total family network measures	Size	Number of family members within the network of the participant with intellectual disability
	Density	An indicator of how close a network is; how many network members support each other on average. Density is defined as the ratio between the number of existing supportive relationships between the family members divided by the total number of possible supportive relationships between the family members
	Arc reciprocity	Proportion of supportive relationships between family members that are reciprocal. The focus of this measure is on the number of supportive relationships that are involved in reciprocal relations, relative to the total number of actual relations
Individual family network measures	In‐degree	Number of relationships in which the person with intellectual disability receives support
	Out‐degree	Number of relationships in which the person with intellectual disability provides support
	Dyad reciprocity	Number of dyads (in which the person with intellectual disability is an actor) with reciprocal relationships, divided by the total number of adjacent dyads (in which the person with intellectual disability is an actor)
	One step outreach centrality	Number of distinct family members within one link of a given person (i.e. how many other people a given person can reach in one step)

#### 2.3.2 Cognitive ability

To check whether a participant met the inclusion criteria of mild intellectual disability, a brief screening IQ‐score, based on standard scores and standard errors, was derived because file scores of the participants were often missing, outdated or obtained using unidentified IQ tests. The subtests “Vocabulary” and “Matrix Reasoning” from the [LANGUAGE] version of the WAIS‐IV (WAIS‐IV‐NL) were used (Wechsler, 2012). These subtests correspond with the subtests of the Wechsler Abbreviated Scale of Intelligence‐II (WASI‐II; Wechsler, 2011). As no [LANGUAGE] version of the WASI‐II was available, the two corresponding WAIS‐IV subtests were used. The raw scores were turned into standardized scores per participant; then, a 95% confidence interval was calculated per subtest with help of the standard measurements of errors. When a participant had a standard score in the intellectual disability range on both subtests (according to the 95% confidence interval), the participant was deemed as having a mild intellectual disability. People with intellectual disabilities often have a varied intelligence profile. Therefore, participants who score on only one subtest in intellectual disability range were also included in the study. Participants who scored above the intellectual disability range on both subtests were excluded.

#### 2.3.3 Well‐being

Subjective well‐being was measured using the Personal Wellbeing Index‐Intellectual Disability (PWI‐ID; Cummins, Lau, Davey, & McGillivray, 2010). Participants with intellectual disability were asked to report their satisfaction with their life as a whole, and on seven life domain items: standard of living, health, achieving in life, relationships, personal safety, community connectedness and future security. Items were rated on a 5‐point response scale, with anchor points of “completely dissatisfied” (1), “neutral” (3) and “completely satisfied” (5). For analysis, individual item scores were used. In previous research, the scale items had item‐total correlations higher than the recommended minimum of 0.30 (McGillivray, Lau, Cummins, & Davey, 2009).

#### 2.3.4 Behavioural and emotional problems

The Adult Behavior Checklist (ABCL; Achenbach & Rescorla, 2003) was used to measure behavioural and emotional problems. This questionnaire examines a broad range of behavioural and emotional problems: anxious/depressed, attention, withdrawal, aggression, somatic complaints, rule‐breaking and intrusive thoughts. Although the ABCL was developed for the general population, a study on the psychometric properties for the use of this instrument with people with intellectual disability has been conducted. Tenneij and Koot (2007) found that the internal consistency coefficient Cronbach’s alpha of the ABCL scales, for people with intellectual disabilities, ranged from 0.69 to 0.95 (mean alpha = 0.84); and inter‐rater reliability, assessed by the intraclass correlation coefficient, ranged from 0.57 to 0.76 (mean = 0.68). In the current study, the present authors used the scores on the eight subscales. Key support workers were asked to rate whether these items were true of the participants over the past 6 months using a 3‐point response scale: “not true” (0), “somewhat or sometimes true” (1) or “very true or often true” (2). Total scores for each scale were converted into T‐scores.

#### 2.3.5 Demographics

Demographic information (participants’ sex, age, living situation and cultural background) was obtained during the interview, or afterwards from the participant’s file (with consent of the participant).

### 2.4 Data analysis

The family network data were entered into Excel and then imported and analysed in UCINET (version 6.623), a software package for the analysis of social network data (Borgatti, Everett, & Freeman, 2002). To identify empirically meaningful family typologies based on the FNM‐ID variables, the present authors used latent class analysis (LCA) in Mplus (Muthén & Muthén, 1998‐2015). LCA is a probabilistic version of traditional non‐hierarchical cluster analysis. The inputs for LCA were the seven social network variables (Table 2) standardized as z‐scores. To identify the ideal number of classes (family typologies), several criteria were used. The first criterion, the measure of parsimony, was the Baysian information criterion (BIC; Kass & Raftery, 1995). A lower BIC value indicates improvement of model fit with k classes relative to a model with k‐1 classes. If the BIC values increase in model k + 1, the preceding number of classes k is most optimal. The second criterion was the classification quality of the model. High average posterior probabilities indicate how well the participant is classified into their class. The entropy measure is a combined index of the posterior probabilities, and high values are preferred with a maximum value of 1. There are no statistical criteria to decide what is low or high. The third criteria were two likelihood ratio tests: Vuong–Lo–Mendell–Rubin likelihood ratio test (VLMR‐LRT) and the adjusted Lo–Mendell–Rubin likelihood ratio test (LMR‐aLRT) (Muthén & Muthén, 1998‐2015), indicating whether the present k‐class solution was better than the foregoing k‐1 class solution. Significant values (p < .05) of the likelihood ratio tests indicated that the present model (k) was superior to the previous (k‐1) model. The fourth criterion was the utility of the classes based on practical and theoretical considerations (Porcu & Giambona, 2017).

After choosing the most ideal number of classes, the stability of the solution was verified by bootstrap sampling (Efron & Tibshirani, 1993). Five thousand bootstrap samples of size N = 137 were generated by sampling with replacement from the original data set. For each bootstrap sample, a LCA was conducted and at the end, all samples are combined to construct confidence intervals (95% CI) for the parameter estimates.

The 95% bootstrap CIs are also used to interpret each of the k classes. A standardized social network variable with a 95% CI including positive as well as negative values is interpreted as having an average level within a class and is denoted by 0. Intervals containing only positive values with mean estimates below .50 are considered as above average and denoted by +. Intervals with only positive values and a mean estimate between .50 and 1.00 are considered as rather high and denoted by ++. A mean estimate above 1.00 with only positive values is seen as high and denoted by +++. Similar criteria are applied for negative intervals.

After the number of classes was identified, the second step was to test differences between the classes with respect to demographic variables, well‐being, and behavioural and emotional problems. Differences across classes with categorical or binary variables were tested with a chi‐square test as described by Lanza, Tan, and Bray (2013) and differences across classes with continuous variables with a chi‐square test as described by Asparouhov and Muthén (2014). Both tests are available in Mplus. An overall chi‐square test was carried out for all four classes together. A significant result was followed by chi‐square tests comparing each two class combinations.

## 3 RESULTS

### 3.1 Latent class analysis

Latent class analyses with 1–5 classes were performed, because after the fifth class the different parameter values became worse. BIC values, entropy values, numbers in each class, posterior probabilities and p‐values for two likelihood ratio tests are displayed in Table 3. With increasing numbers of classes, the BIC value decreased. However, there was no turning point with an increasing BIC value. Based on this criterion, it was not possible to decide the optimal number of classes, but the decrease in BIC value is very low from class 4 to class 5. The entropy measure was highest with k = 3 classes. In fact, the entropy values are high for all class solutions (>0.95), so this criterion is not useful for deciding the number of classes. Both likelihood ratio tests indicated that a 2‐class solution would be better than a 3‐class solution. In the 4‐class model, a distinctive meaningful group emerged based on face validity. The two classes in the 2‐class solution resembled the first two classes of the 3‐, 4‐ and 5‐class solutions. The third class of the 3‐class solution resembled the third class in the following solutions. The fourth class in the 4‐class solution resembled the fourth classes in the 5‐class solution. The fifth class in the 5‐class solution did not resemble the first four classes.

**TABLE 3:** Latent class models with up to five classes

LCA	Classes	n	Posterior probability	VLMR‐LRT *p* value	LMR‐aLRT *p* value
Class = 1	1	137	1.00		
BIC = 2783					
Classes = 2	1	88	.98	.000	.000
BIC = 2473	2	49	.99		
Entropy = 0.966					
Classes = 3	1	86	.99	.059	.064
BIC = 2395	2	35	.99		
Entropy = 0.972	3	16	.98		
Classes = 4	1	79	.99	.185	.191
BIC = 2359	2	35	.99		
Entropy = 0.970	3	14	.99		
	4	9	.97		
Classes = 5	1	68	.98	.393	.403
BIC = 2340	2	22	.94		
Entropy = 0.958	3	14	.98		
	4	8	.99		
	5	25	.98		

Based on statistical criteria, the 4‐class solution would be better than the 5‐class solution because the posterior probabilities of the 5‐class solution are even lower, and there is only a small decrease in the BIC value. However, based on face validity, the 4‐class solution would also be the best fit because four characteristic groups emerged. The fifth class did not show new differential characteristics and the number of average scores on the network measures increased compared to the other classes. The entropy value for the 4‐class solution was high (.958) with high posterior probabilities (.973 ‐ .988) indicating that the participants were correctly classified in their classes. Based on the combination of all criteria used, a 4‐class solution was chosen.

### 3.2 Description of the Four Classes

To be able to describe the four different classes and depict them with illustrative graphs (NetDraw, Borgatti, 2002), the raw mean scores for each of the classes on the seven social network variables were calculated (Table 4).

**TABLE 4:** Means scores, 95% bootstrap confidence intervals and typology of four family network classes

	Class 1 N = 79				Class 2 N = 35				Class 3 N = 14				Class 4 N = 9			
	*M (SD)*	95% BCI		Score categorization	*M (SD)*	95% BCI		Score categorization	*M (SD)*	95% BCI		Score categorization	*M (SD)*	95% BCI		Score categorization
Size	6.37 (3.17)	−0.35	0.02	0	4.57 (1.70)	−0.67	−0.37	‐‐	9.43 (4.05)	−.01	1.13	0	20.56 (4.42)	1.01	3.46	+++
Density	0.25 (0.14)	−0.58	−0.31	‐	0.61 (0.22)	0.66	1.43	+++	0.49 (0.21)	−.01	1.09	0	0.09 (0.05)	−1.24	−0.54	‐‐‐
Arc reciprocity	0.34 (0.26)	−0.70	−0.28	‐	0.78 (0.22)	0.59	1.16	++	0.69 (0.14)	.30	.88	++	0.39 (0.24)	−0.97	0.17	0
Dyad reciprocity	0.11 (0.21)	−0.72	−0.35	‐‐	0.66 (0.28)	0.75	1.45	+++	0.44 (0.18)	.19	.83	+	0.16 (0.18)	−0.74	0.16	0
One step outreach centrality	0.10 (0.16)	−0.79	−0.53	‐‐	0.76 (0.24)	0.82	1.34	+++	0.86 (0.15)	1.08	1.59	+++	0.17 (0.17)	−0.80	0.01	0
In‐degree	1.97 (1.47)	−0.44	0.03	0	2.23 (1.33)	−0.36	0.28	0	4.57 (1.40)	.88	1.94	+++	2.22 (1.20)	−0.54	1.06	0
Out‐degree	0.67 (0.98)	−0.62	−0.42	‐‐	2.69 (1.28)	0.01	0.41	+	9.07 (2.56)	1.88	3.31	+++	3.00 (3.04)	−0.45	1.65	0

Note

M = mean; 95% BCI = 95% bootstrap confidence interval; score categorization = interpretation mean and 95% CI.

+++ = high, ++ = rather high, + = above average, 0 = average, ‐ = below average, ‐‐ = rather low, ‐‐‐ = low.

To give a global impression of what the classes look like, the descriptions of the four classes are supported by example graphs of the perceived networks for individual “typical” participants. These four participants were selected as examples because they had individual scores that approached the mean scores on the social network measures for their class.

**FIGURE 1:**
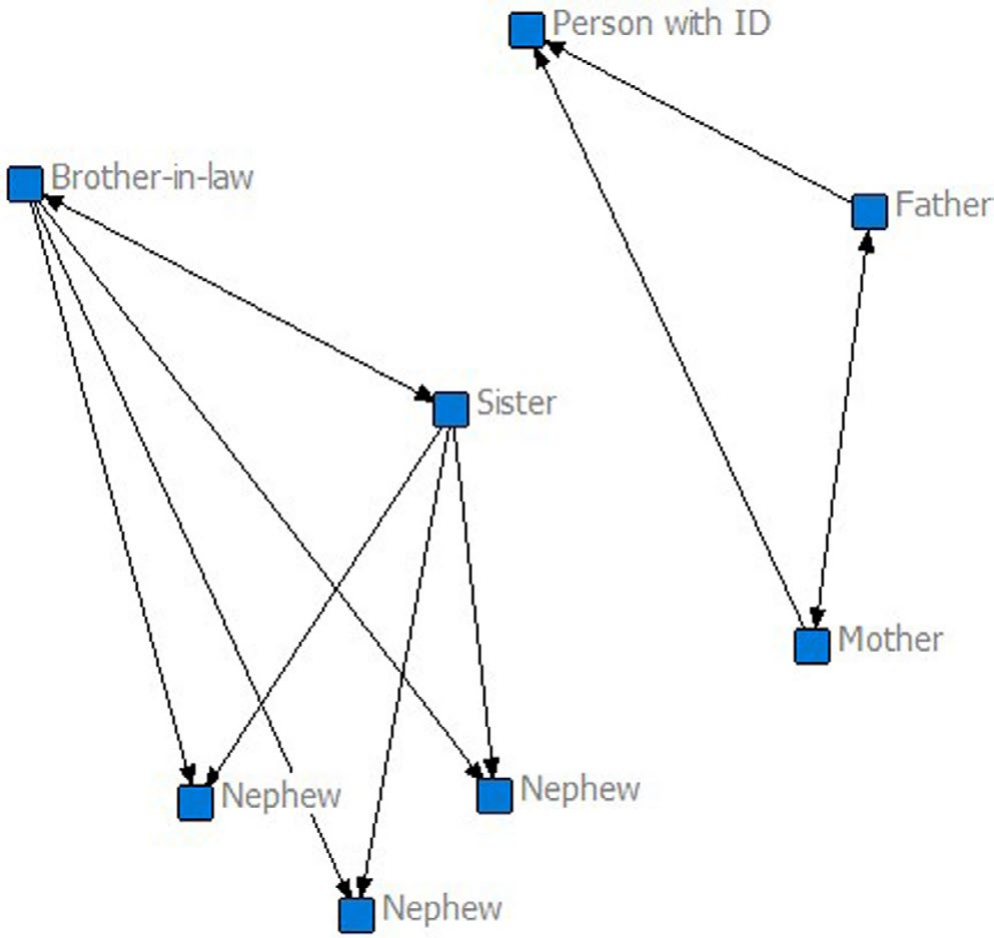
Example of a family network of a participant in class 1

Class 1 (*n* = 79) can be described as an overall small network (mean 6.37 people), with a small number of supportive relationships (*M* = 1.97, *SD* = 1.47), and the person with intellectual disability rarely provides support (*M* = 0.67, *SD* = 0.98). The person with intellectual disability has very little reciprocal support (*M* = 0.11, *SD* = 0.21), and they cannot reach many family members in their network directly (*M* = 0.10, *SD* = 0.16). Figure 1 is an example of a participant of class 1 who only gets support from his parents. His sister and brother‐in‐law are also listed in his family network, but they are not providing or getting any emotional support from the participant. This network characterizes more than one half of participants. It represents a small network and impoverished experience in terms of family‐based social capital.

**FIGURE 2:**
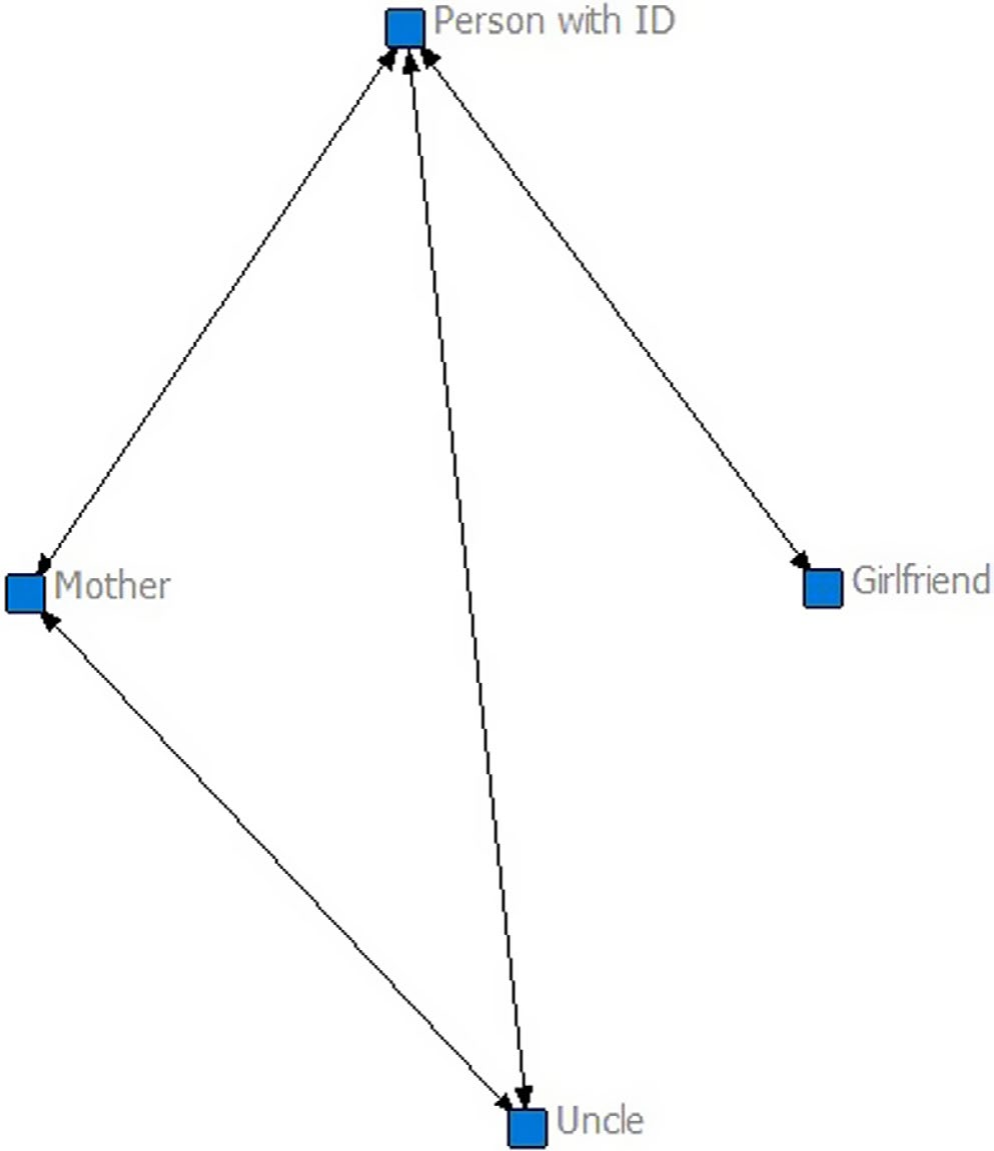
Example of a family network of a participant in class 2

Class 2 (*n* = 35) represents small networks (*M* = 4.57, *SD* = 1.70) but scores high on density (*M* = 0.61, *SD* = 0.22) and reciprocity for the whole network (*M* = 0.78, *SD* = 0.22). The person with intellectual disability receives and gives somewhat more support (*M* = 2.23, *SD* = 1.33; *M = *2.69, *SD* = 1.28) compared to participants in class 1. Participants in class 2 can relatively easily reach most other people in their network (*M* = 0.76, *SD* = 0.24). The example shows a small but dense family network. The participant reported that he is supporting every family member in his network and also receives emotional support from all of them. Overall, this class seems to describe a small but supportive family network (Figure 2).

**FIGURE 3:**
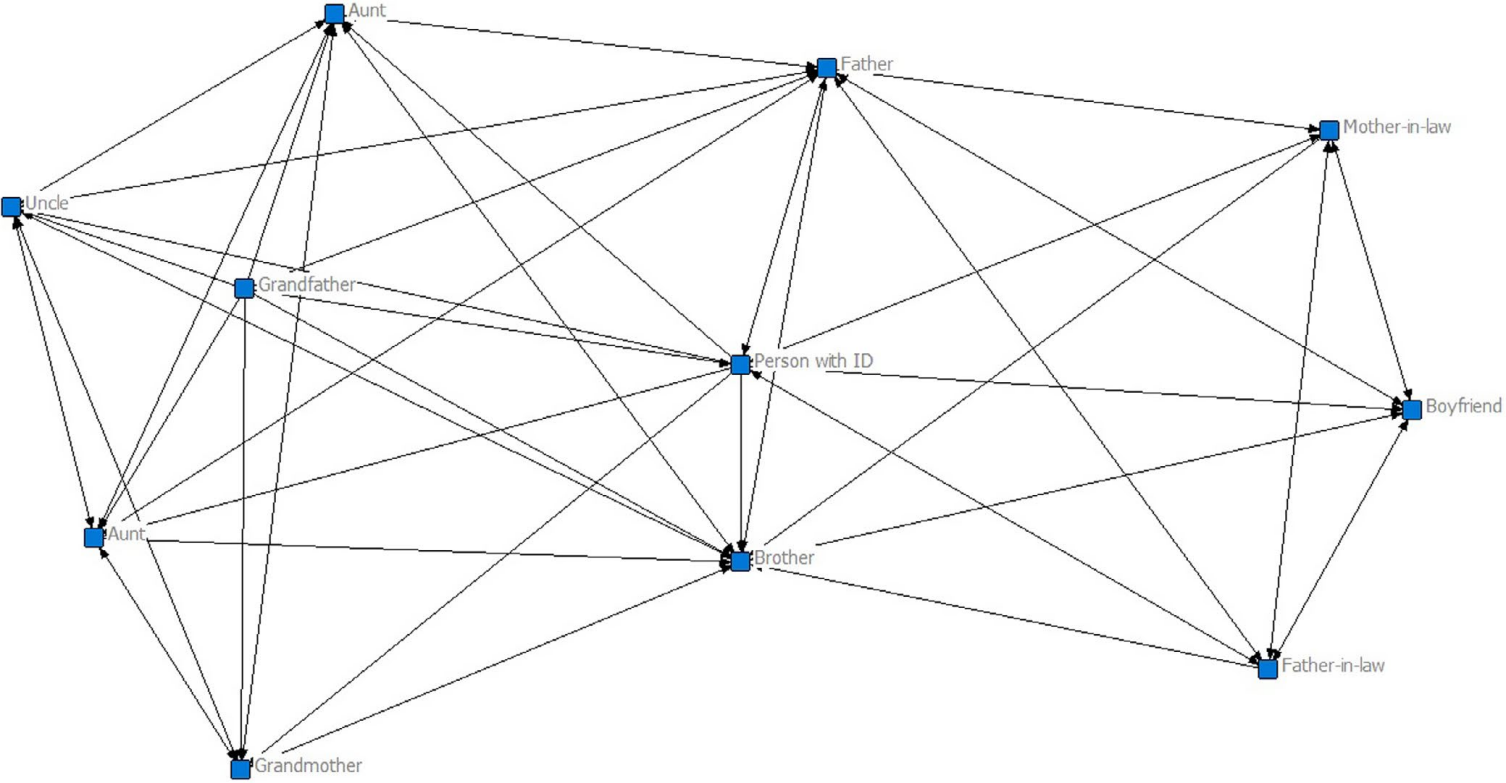
Example of a family network of a participant in class 3

Class 3 (*n* = 14) applies to a small number of people. The network has a moderate size (*M* = 9.43, *SD* = 4.05) and density (*M* = 0.49, *SD* = 0.21). It also has the largest number of supportive people (*M* = 4.57, *SD* = 1.40) and people who are supported by the person with an intellectual disability (*M* = 9.07 *SD* = 2.56). Individuals with an intellectual disability also have a direct connection to most people in their network (*M* = .86, *SD* = .15). Hence, the graph shows that the person with intellectual disabilities is centrally placed in his network. Overall, this class represents a large type of family with close relationships that include the person with an intellectual disability (Figure 3).

**FIGURE 4:**
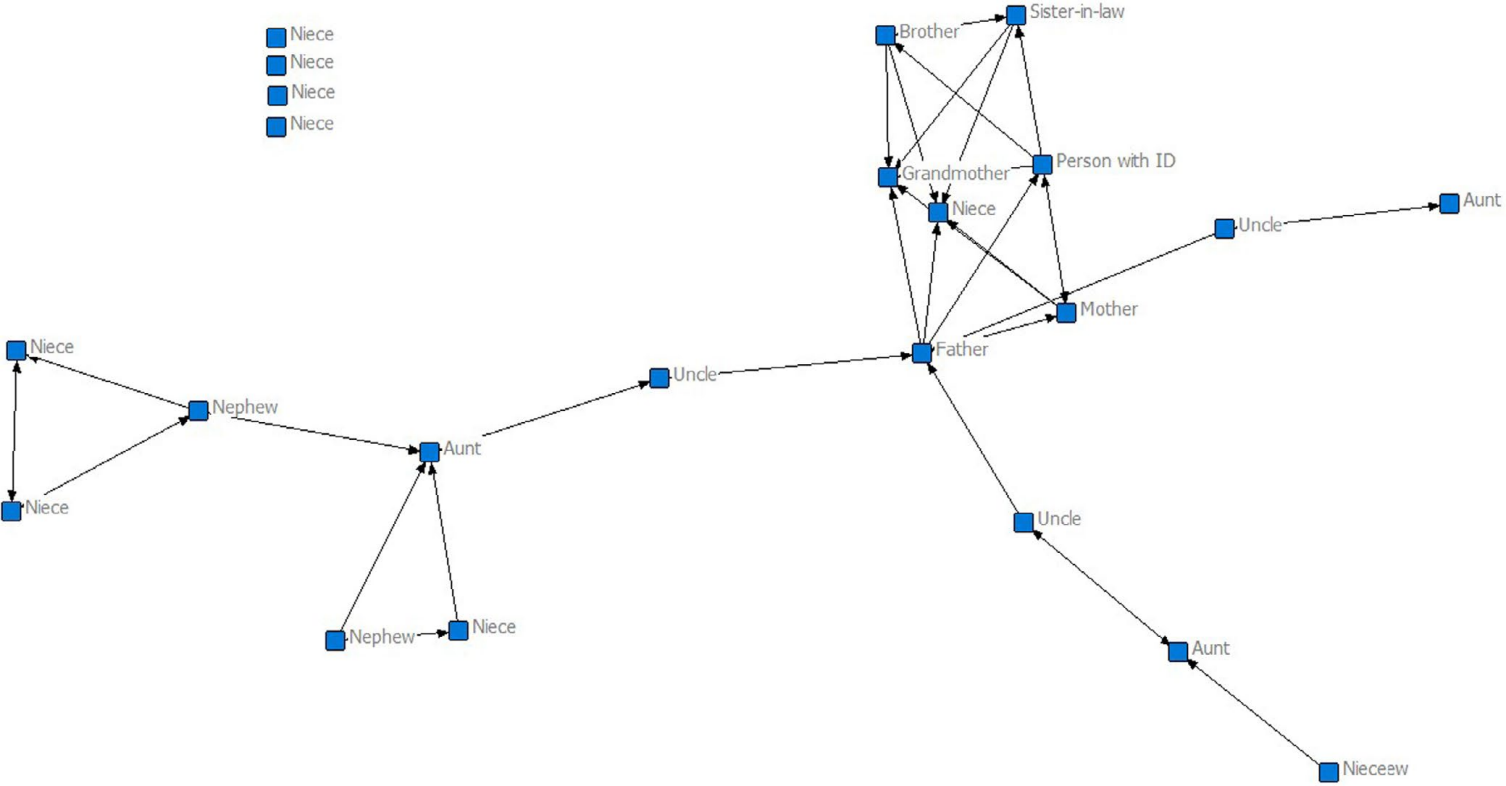
Example of a family network of a participant in class 4

Class 4 (*n* = 9) applies to a very small number of people but was reasonably stable across the multiple solutions. Class 4 is represented by a large family (*M* = 20.56, *SD* = 4.42) with fewer connections (*M* = 0.09, *SD* = 0.05) and limited reciprocity (*M* = 0.39, *SD* = 0.24). The person with intellectual disability is supported by a small proportion of the network (*M* = 2.22, *SD = *1.20) and, in turn, supports few family members (*M* = 3.00, *SD* = 3.04). The individual example shown in Figure 4 has a substantial family network. However, he is only a member of a small sub‐section of the network. In this sub‐section, he enjoys mutually supportive relationships with family members. It is notable that four nieces who are listed do not get any emotional support from the other family members. This is a large type of family network, whose members do not appear to enjoy close relationships. The person with an intellectual disability only view themselves as having connections with a small part of the family network (they can reach on average .17 of them in one step).

### 3.3 Comparisons across classes

To test differences across the four classes, the present authors used a chi‐square test for categorical/binary outcomes (Lanza et al., 2013) and a chi‐square test for continuous outcomes (Asparouhov & Muthén, 2014), as presented in Table 5. Significant differences across the four classes were found for cultural background, χ^2^(3) = 12.08, *p* = .007, well‐being within personal relationships, χ^2^(3) = 14.79, *p* = .002, community connectedness, χ^2^(3)= 8.16, *p* = .043, thought problems, χ^2^(3) = 8.22, *p* = .042, rule‐breaking behaviour, χ^2^(3) = 8.19, *p* = .042, and intrusive behaviour, χ^2^(3) = 10.32, *p* = .016.

**TABLE 5:** Centrality measures (proportions and means) and standard deviations for demographic, well‐being and behavioural and emotional problem variables with test results for differences between classes

	Class 1	Class 2	Class 3	Class 4	Overall chi‐square test		Post hoc chi‐square test		Classes
	(*n* = 79)	(*n* = 35)	(*n* = 14)	(*n* = 9)	χ^2^(3)	*p*	χ^2^(1)	*p*	differing
Sex (proportion female)	0.40 (0.49)	0.49 (0.53)	0.43 (0.52)	0.58 (0.52)	1.32	.724			
Living situation (proportion living individually)	0.32 (0.45)	0.46 (0.52)	0.35 (0.51)	0.11 (0.32)	6.21	.102			
Residential (proportion with residential facility)	0.13 (0.32)	0.17 (0.37)	0.36 (0.49)	0.11 (0.34)	3.32	.357			
Cultural background (proportion [NAME COUNTRY])	0.91 (0.43)	0.92 (0.30)	1.00 (0.00)	0.89 (0.32)	12.08	.007	7.20	.007	3 vs. 4
Age	28.57 (6.25)	28.57 (6.13)	23.22 (8.51)	31.08 (5.93)	7.28	.064			
Well‐being—life as a whole	3.92 (0.85)	3.95 (0.92)	4.07 (0.62)	4.10 (1.19)	.55	.908			
Well‐being—standard of living	4.38 (0.74)	4.28 (0.72)	4.36 (0.73)	3.36 (1.37)	4.72	.194			
Well‐being—health	3.85 (1.05)	3.89 (0.86)	3.78 (0.95)	3.88 (1.30)	.13	.988			
Well‐being—achieving in life	4.20 (0.69)	4.26 (0.82)	4.07 (0.71)	4.11 (0.59)	.81	.847			
Well‐being—personal relationships	4.12 (0.79)	4.41 (0.66)	4.72 (0.46)	4.32 (1.01)	14.79	.002	14.55	.000	1 vs. 3
Well‐being—personal safety	3.91 (0.85)	3.93 (1.02)	4.21 (0.88)	3.89 (1.10)	1.38	.710			
Well‐being—community connectedness	3.99 (0.87)	4.35 (0.59)	4.21 (0.78)	4.46 (0.51)	8.16	.043	6.00 5.28	.014 .022	1 vs. 2 1 vs. 4
Well‐being—future security	3.97 (0.79)	4.06 (0.74)	4.21 (0.95)	4.08 (0.86)	.97	.809			
Anxious/depressed	9.43 (5.60)	10.37 (7.67)	10.69 (5.92)	12.23 (6.98)	1.89	.596			
Withdrawn	5.81 (3.73)	5.49 (4.15)	6.30 (4.37)	5.42 (3.64)	.45	.929			
Somatic complaints	3.08 (4.04)	5.16 (4.22)	3.03 (2.31)	5.59 (5.23)	6.69	.082			
Thought problems	2.52 (2.18)	2.92 (2.99)	3.14 (3.15)	6.46 (4.18)	8.22	.042	7.67 5.62 4.14	.006 .018 .042	1 vs. 4 2 vs. 4 3 vs. 4
Attention problems	13.23 (5.70)	13.61 (7.05)	17.33 (6.01)	16.15 (6.29)	6.81	.078			
Aggressive behaviour	9.06 (7.03)	10.45 (7.24)	11.80 (6.72)	10.74 (7.99)	2.48	.478			
Rule‐breaking behaviour	5.37 (7.87)	6.03 (5.34)	10.20 (5.65)	8.18 (13.08)	8.19	.042	7.70 5.57	.006 .018	1 vs. 3 2 vs. 3
Intrusive	3.30 (2.80)	4.22 (3.18)	5.58 (2.64)	4.70 (2.47)	10.32	.016	8.55	.003	1 vs. 3

Note

Only significant overall chi‐square tests with *p* < .05 were followed with chi‐square post hoc tests.

For the outcomes with an overall significant difference across classes, centrality measures (proportions or means) for each class were calculated and post hoc chi‐square tests used to examine which classes were different from each other.

The most distinct class is class 3. This class only has participants from a [NAME COUNTRY] cultural background, and they had the highest well‐being scores for their personal relationships and the highest scores for rule‐breaking and intrusive behaviour. Class 4 is also notably different. Participants in this class had the highest scores for thought problems and the highest scores for community connectedness. Finally, participants in class 1 were the least satisfied with their community connectedness and personal relatedness. Participants in this class, on the other hand, displayed the lowest levels of behavioural and emotional problems.

## 4 DISCUSSION

The present authors used social capital‐informed research methods to characterize the family network typologies of people with mild intellectual disability, using their self‐reports about family members’ emotional support. Through latent class analyses, four family network typologies were identified based on seven self‐reported social network measures. The four distinguishable family network typologies show that people with intellectual disability have a variety of family contexts with distinct social capital. Class 2 and class 3 appear to be the most supportive family networks, in which the person with mild intellectual disability is part of a close (reciprocally supportive) family group. In contrast, participants in classes 1 and 4 may experience less family‐based social capital. Participants in class 1 had a small family network in terms of both size and support. They are also the least satisfied with their personal relationships and their community connectedness. Class 4 may represent large family networks but they are not perceived to be close, although the participants in this class do score well on community connectedness.

The insights obtained from analysing these different types of perceived family groups might have practical use for professionals when looking at ways to strengthen, maintain or expanding the social capital of people with intellectual disabilities. For example, in the case of class 1, expanding the size of the network and the (reciprocal) supportive relationships might have a positive influence on their subjective well‐being and mental health. Participants in class 4 could take more advantage of their available connections to become more connected with their extensive family network. However, caution is needed here because the distance might be there for a reason. For example, family relationships could be distant due to a family member being abusive or difficult family relationship; or certain characteristics or actions of the individual with intellectual disabilities may have influenced the nature of their relationship (Greenberg, Seltzer, Hong, & Orsmond, 2006).

Although classes 2 and 3 are both supportive family typologies, participants in class 3 seem to be happier with their personal relationships. A possible explanation might be linked to their extended family networks and reciprocal relationships. The reciprocal nature of relationships can improve and strengthen the connections (Baumeister & Leary, 1995) and have a positive effect on the self‐worth of people with intellectual disability (Milner & Kelly, 2009). In turn, this might contribute to the high score for well‐being in terms of personal relationships. At the same time, participants in class 3 also scored highest on the subscales rule‐breaking and intrusive behaviour of the ABCL. Typically, people with intellectual disability who have behavioural and emotional problems are among the most disadvantaged and socially excluded in society (Emerson, 2001). The results for class 3 seem at odds with earlier research. There may be at least two possible hypotheses for this result. First, the inflated self‐perceptions of people with behavioural problems have been attributed to an illusory positive bias (Barry, Kerig, Stellwagen, & Barry, 2011). Another potential explanation could be that people who are more aggressive are less passive and more demanding, and so are better at maintaining relationships with family members. These hypotheses, and others, could be explored in future in‐depth research with people with intellectual disabilities who have families matching the class 3 typology.

Limitations of the current study should be mentioned. First, the present authors focused on emotional support because it has been found to be a stronger predictor for physical and mental health‐related outcomes (Berkman, 1995; Thoits, 1995). However, different results may be obtained if the focus is on instrumental support. Second, no rating of the quality of emotional support was included. Quality of social relations may have an impact on well‐being. Positive aspects of supportive relationships appear to provide a sense of security, increasing individuals’ positive feelings about themselves and their world (Antonucci, 2001). Future research could replicate the current methods with a focus on instrumental support and the quality of emotional support—to examine the replicability of the typologies identified. Third, there were some limitations concerning sampling and recruitment. Only 42.4% (*n* = 150) of the participants who were initially identified agreed to take part in the study meaning that the resulting sample was unlikely to have been representative of the population studied. Unfortunately, no data were available for the non‐respondents. Therefore, it was not possible to quantify biases in the sample selection. The present authors only included participants who lived apart from their family in long‐term care and who were supported by staff. Their distance from family members and the nature of their support may have had a significant impact on their perceptions of family. In future research, it would be important to examine family typologies in populations living in different contexts, and cultures, and with more representative samples.

The current study was exploratory, and yielded insights about family network typologies of people with mild intellectual disability from their own perspectives. The findings showed that their social capital is low on average but that there is some variability. In terms of practical implications, these findings suggest that people with intellectual disability have different support needs in terms of strengthening or extending their social capital.
